# Structural Identification of Impurities in Pioglitazone Hydrochloride Preparations by 2D-UHPLC-Q-Exactive Orbitrap HRMS and Their Toxicity Prediction

**DOI:** 10.1155/2023/2096521

**Published:** 2023-10-17

**Authors:** Dandan Zhang, Weijian Wang, Haiyun Zhao, Song Wang, Mingyan Yu, Dongmei Zhang, Wenkun Liu, Qiangsheng Xie, Dejun Chen

**Affiliations:** Shandong Institute for Food and Drug Control, Shandong Research Center of Engineering and Technology for Consistency Evaluation of Generic Drugs, NMPA Key Laboratory for Research and Evaluation of Generic Drugs, Public Service Platforms for Industrial Technology, Jinan, China

## Abstract

Pharmaceutical companies and regulatory agencies have more and more concerns for impurities in pharmaceuticals and their toxicity. In this work, heart-cutting two-dimensional ultrahigh-performance liquid chromatography (2D-UHPLC) in combination with high-resolution mass spectrometry (HRMS) was used, setting HRMS as positive mode of electrospray ionization to identify five impurities in pioglitazone hydrochloride preparations. With the heart-cutting 2D-UHPLC and online desalting technique, the structures of five impurities were deduced in an analysis of MS^n^ data. And three of them, Impurity-2, Impurity-3, and Impurity-5, have never been reported before. The fragmentation patterns of five impurities were proposed on a basis of accurate mass and fragment ions in this study. Since the toxicity of impurities is relevant to their structures, toxicology of all five impurities was predicted by three software tools, and the result showed that these compounds have good safety profile.

## 1. Introduction

As a thiazolidinedione insulin sensitizer, pioglitazone can activate peroxisome proliferator-activated receptor-gamma (PPAR-*γ*), which is distributed predominantly in the adipose tissue, skeletal muscle, and liver [1–3]. PPAR-*γ* regulates lipogenesis, fatty acid storage, insulin sensitivity, and glucose metabolism [4]. Pioglitazone, a potent and selective PPAR-*γ* agonist, can improve insulin sensitivity and enhance hyperglycemia [5]; thus, it has been widely used for treatment on type 2 diabetes mellitus (T2DM) [6]. Pioglitazone hydrochloride manufactured by Takeda Pharmaceutical Company Limited was approved in 1999 in the US and marked as ACTOS® [7].

The impurities of drug product have negative impaction for its safety and its efficacy. This study focused on the related substances of pioglitazone hydrochloride preparations from the surveillance sampling of Shandong Medical Product Administration. The method described in European Pharmacopeia (EP) 10.0 Pioglitazone Hydrochloride was applied to analyze the impurities in inspected samples. Excepting eight identified impurities, five unidentified impurities were detected with high frequency in 54 batches of inspected pioglitazone hydrochloride preparations that were produced by 13 manufacturers.

UHPLC-Q-exactive orbitrap HRMS is widely used for the detection and identification of impurities, as UHPLC (ultrahigh-performance liquid chromatography) provides a rapid and effective separation, while HRMS (high-resolution mass spectrometry) offers accurate mass and fragment ions, which are beneficial for structural elucidation [8, 9]. It is known that all LC-MS methods are based on a volatile salt in mobile phase. Although ammonium acetate, used for separation and determination of the related substances in EP 10.0 for pioglitazone hydrochloride, is a volatile salt, its high concentration leads to strong ion suppression effect which may decrease the MS sensitivity of the impurities. Therefore, no peak was detected in the total ion chromatogram (TIC) when target impurities were directly injected into MS without desalting. Over the past decades, two-dimensional liquid chromatography (2D-LC) technique has been developing rapidly and been applied extensively in the field of pharmaceutical analysis [10–12]. Generally, 2D-LC can be classified into two types: comprehensive two-dimensional liquid chromatography which transports continuous stream of effluent from 1D column into 2D column, and heart-cutting two-dimensional liquid chromatography which transfers targeted portion (the peak of aimed impurity) of the 1D effluent into the 2D column. For heart-cutting 2D-LC, the first chromatographic dimension is utilized to trap the targeted impurities into quantitative loop by a switching valve, while the second chromatographic dimension serves as an online desalting segment which can remove the nonvolatile salt or high concentration of volatile salt from the first dimension with a low concentration of volatile mobile phase [10]. And the present study employed a heart-cutting 2D-LC coupled with high-resolution mass spectrometry (HRMS) to characterize the structures of five unidentified impurities, and three of them was never been reported before.

Assessing the biological toxicity of impurities is beneficial for the quality control of drug products. While animal model for toxicity accessing has many constrains, researchers focus on computational methods. Since impurity toxicity is closely relevant to its molecular structure, structure-activity relationships (SARs) have been normally used in the pharmaceutical industry to estimate their toxicity by computer [13]. In this work, ADMET Predictor™ 8.0, Derek Nexus 5.0.1 (knowledge-based), and Sarah Nexus 2.0.1 (statistics-based) were employed to evaluate the toxicity of target impurities based on the speculated structures.

## 2. Experimental Methods

### 2.1. Samples and Reagents

54 batches of investigated pioglitazone hydrochloride preparations (including pioglitazone hydrochloride tablets and pioglitazone hydrochloride capsules) were obtained from the surveillance sampling of Shandong Medical Product Administration. The reference substance pioglitazone hydrochloride (purity 100.0%, batch number 100634–201703) was purchased from the National Institute for Food and Drug Control (Beijing, China). Ammonium acetate (purity 98.3%) and acetonitrile (HPLC grade) were supplied by Fisher Chemical (USA). Water used for all analyses was purchased from Watsons (China).

### 2.2. Instrumentation

The Agilent 1260 Infinity II HPLC (Agilent Technologies, USA) was used for screening impurities for investigation. The UHPLC-HRMS System (Thermo Fisher Scientific, Germany) consists of ultimate 3000 pump, autosampler, column compartment, and orbitrap high-resolution mass spectrometer. Tune 2.9 software (Thermo Fisher Scientific, USA) was used to control the mass spectrometer. XCalibur 4.0 software (Thermo Fisher Scientific, USA) was used for instrument control and data processing. Compound Discoverer 3.1 was adopted to analyze the molecular formulas of investigated impurities. Mass Frontier 7.0 software was utilized to analyze the fragmentation mechanism of mass spectrometry. Chromatographic separation was achieved by an Inertsil ODS-3 C18 column (250 mm*∗*4.6 mm, 5 mm) (Thermo Fisher Scientific, USA). The desalting was achieved by an Inertsil ODS-SP C18 column (150 mm*∗*4.6 mm, 5 mm) (Thermo Fisher Scientific, USA). The centrifugation was performed on a 5804R refrigerated centrifuge (Eppendorf, Germany). The ultrasonic process was operated on a KQ-500DE Thermostat Ultrasonic Instrument (Kunshan, China). MS105DU Analytical Balance (Mettler Toledo, Switzerland) was used to weight. ADMET PredictorTM 8.0 software (Simulations plus Inc., USA), Derek Nexus 5.0.1 software (Lhasa Limited, UK), and Sarah Nexus 2.0.1 software (Lhasa Limited, UK) were employed to predict the toxicity of impurities.

### 2.3. Sample Preparation

54 batches of pioglitazone hydrochloride preparations were in the form of tablets or capsules. For tablets, 20 tablets were grinded into homogeneous powder, whereas for capsules, the shells of 20 capsules were removed and then the powder was mixed. The powder (containing about 20 mg pioglitazone) was accurately weighed and transferred into a 100 mL volumetric flask, followed by adding 20 mL of methanol to dissolve by sonication. Then, the sample was diluted with mobile phase to volume and mixed well, followed by centrifugation at 8000 rpm for 10 min. Finally, 20.0 *μ*L of the top supernatant was taken for screening target impurities, and 100.0 *μ*L of the supernatant was injected for online two-dimensional UHPLC-HRMS analysis.

### 2.4. Chromatographic Conditions

First-dimensional separation conditions are as follows: chromatographic column: Inertsil ODS-3 C18 column (4.6 × 250 mm, 5 *μ*m); column temperature: 35°C; mobile phase: 0.1 mol/L solution of ammonium acetate, acetonitrile, and glacial acetic acid (25 : 25 : 1, V/V/V); detection wavelength: 269 nm; flow rate: 0.7 mL/min. This first-dimensional separation condition was also performed on Agilent 1260 Infinity II HPLC to analyze 54 batches of samples, subsequently screening targeted impurities.

Two-dimensional separation conditions are as follows: chromatographic column: Inertsil ODS-SP C18 column (4.6 × 150 mm, 5 *μ*m); column temperature: 35°C; mobile phase: 5 mmol/L solution of ammonium acetate (A) and acetonitrile (B); flow rate: 0.3 mL/min; the gradient elution program is shown in Table 1.

Mass spectrometry conditions: The Q-exactive orbitrap HRMS was equipped with an HESI ion source and was operated in a positive mode. The ionization parameters were set as follows: spray voltage of 3.8 kV, capillary temperature of 320°C, and vaporizer temperature of 250°C, and the sheath gas, auxiliary gas, and S-lens RF levels were set at 40 arb (arbitrary units), 10 arb, and 50 arb, respectively. Spray stabilization and collision-induced dissociation in the higher energy collision dissociation (HCD) cell adopted high purity nitrogen gas (purity 99.9%). The MS analysis was operated in full MS/dd-MS^2^ (data-dependent MS^2^) mode. The selected scan range of full MS scan was from *m/z* 50 to 750, and the resolution was 70,000. For the dd-MS^2^ scan, the mass resolution was set to 17,500; AGC target was set at 1e5, maximum injection time (IT) was set at 50 ms, and stepped NCE was set to 10, 20, and 30.

### 2.5. 2D-UHPLC System and Online Desalting Procedure

**Figure 1:**
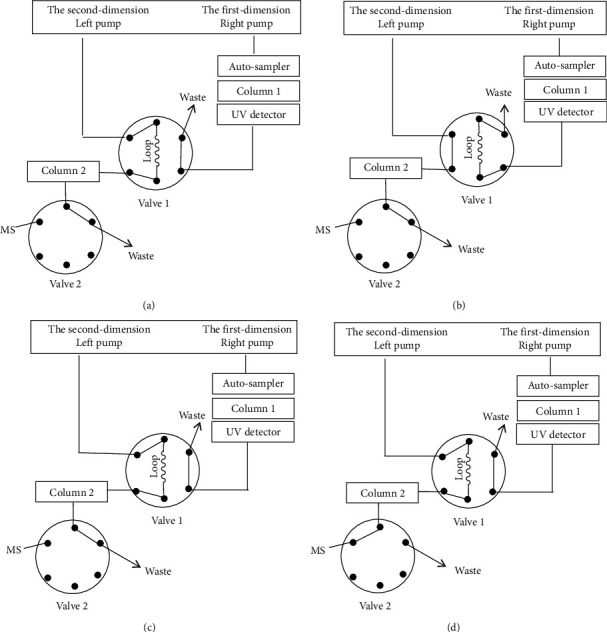
Column switching procedures showing online desalting system.

2D-UHPLC system and online desalting procedure are illustrated in Figures 1(a)–1(d). The trapping of target impurities and online desalting was achieved by valve switching. A loop of 500 *μ*L was equipped on Valve 1 (Figure 1), which was used to collect target impurities eluting from the first dimension. In the beginning of the analysis, the first dimension with the high concentration salt was used to separate all impurities, while the second dimension only started to equilibrate Column 2 (Figure 1(a)). When the peak of target impurity was detected at the first-dimensional UV detector, the target impurity was transferred into a loop of 500 *μ*L by Valve 1 switching (Figure 1(b)). After the trapping of target impurity was finished, Valve 1 was switched back (Figure 1(c)). Then, the mobile phase from the second dimension transferred the impurity in the loop into Column 2. This step sustained for 8 min, and the high concentration salt was flushed out into waste. After that, Valve 2 was switched (Figure 1(d)), the target impurity was introduced into MS.

### 2.6. Toxicity Prediction

Based on the speculated structures, Gastroplus 9.0 ADMET Predictor™ 8.0 software was used to predict the toxicity of target impurities, while Derek Nexus 5.0.1 (knowledge-based) software and Sarah Nexus 2.0.1 (statistics-based) software were applied to evaluate the genotoxicity.

## 3. Results and Discussion

### 3.1. Selection of Target Impurities

**Figure 2:**
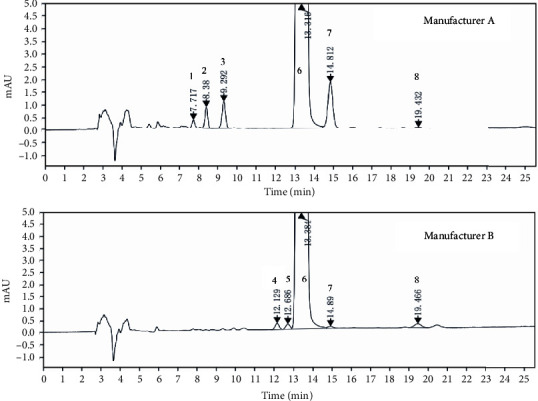
HPLC chromatograms of pioglitazone hydrochloride preparations (detection wavelength 269 nm; peak 6: pioglitazone; peak 1-peak 5: unidentified impurities, target impurities in this study, named as Impurity-1 to Impurity-5, respectively; peak 7: identified impurity, pioglitazone aldehyde; peak 8: identified impurity, impurity A listed in European pharmacopeia 10.0).

The method for detecting related substances in pioglitazone hydrochloride preparations was performed referring to the analytical method of European Pharmacopoeia 10.0 Edition for pioglitazone hydrochloride, as described under Section 2.4. Five unidentified impurities were detected with high frequency in 54 batches of pioglitazone hydrochloride preparations, especially with high-level in 2 batches of products from 2 manufacturers (Figure 2). Therefore, their structures became the objective of this study.

### 3.2. Identification of Target Impurities

**Figure 3:**
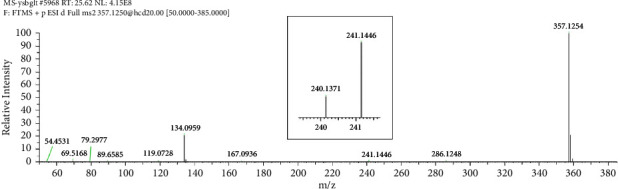
MS^2^ spectra of *m/z* 357.1254.

**Figure 4:**
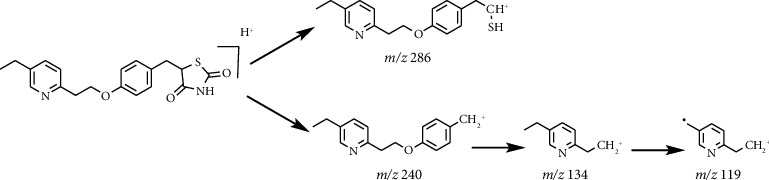
Fragmentation patterns of [M + H]^+^ for pioglitazone.

Fragmentation pattern of pioglitazone is beneficial to elucidate the structures of target impurities. HRMS analysis of pioglitazone showed a protonated molecular ion peak at *m/z* 357.1260 [M + H]^+^ corresponding to molecular formula C_19_H_20_N_2_O_3_S (exact mass: 356.1189).  Figure 3 shows MS^2^ spectrum of *m/z* 357.1254.  Figure 4 shows fragmentation patterns of [M + H]^+^ for pioglitazone. The cleavage product at *m/z* 240 is a loss of 2,4-thiazolodinedione (-C_3_H_3_NO_2_S) from [M + H]^+^, and *m/z* 286 can be attributed to the ring opening of 2,4-thiazolodinedione to remove oximide (-C_2_HNO_2_). The product ions at *m/z* 134, 119 were the characteristic product ions derived from the cleavage of ethoxy phenyl ether bond.

**Figure 5:**
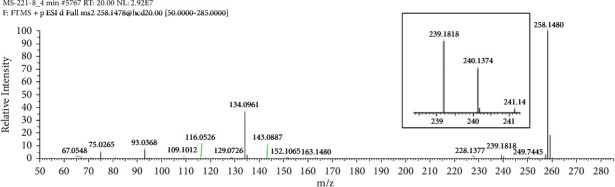
MS^2^ spectra of *m/z* 258.1480.

**Figure 6:**
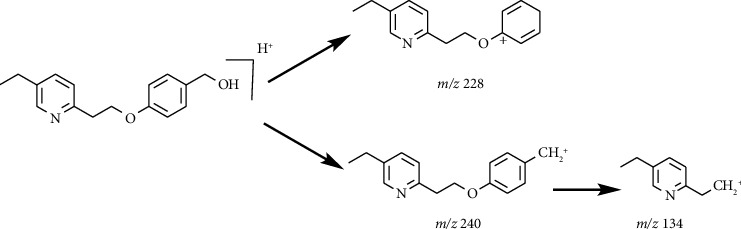
Fragmentation patterns of [M + H]^+^ for Impurity-1.

A protonated molecular ion peak at *m/z* 258.1480 [M + H]^+^ observed in the HRMS spectrum of Impurity-1 was matched to the molecular formula C_16_H_19_NO_2_ (exact mass: 257.1410).  Figure 5 shows the MS^2^ spectrum of *m/z* 258.1480. Characteristic product ions of Impurity-1 at *m/z* 134 and 240 indicated that its structure is unchanged compared with pioglitazone except 2,4-thiazolodinedione unit. The characteristic product ions at *m/z* 240 and 228 were corresponding to the loss of H_2_O, CH_2_=O from [M + H]^+^, respectively, indicating the presence of hydroxymethyl group (-CH_2_OH) in the structure of Impurity-1. The structure of Impurity-1 is shown in Table 2.  Figure 6 shows fragmentation patterns of [M + H]^+^ for Impurity-1.

**Figure 7:**
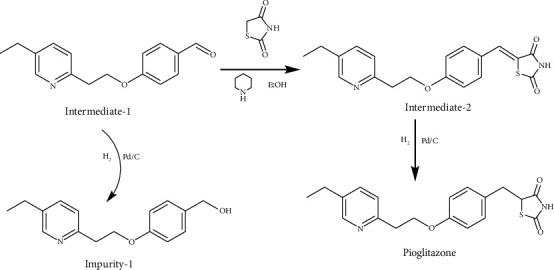
The formation mechanism for Impurity-1.

Impurity-1 is a process impurity. As shown in Figure 7, Intermediate-1 reacted with 2,4-thiazolidinedione to form Intermediate-2 via Knoevenagel condensation, and then Intermediate-2 was reduced to pioglitazone. Meanwhile, the remaining Intermediate-1 could be reduced to Impurity-1.

**Figure 8:**
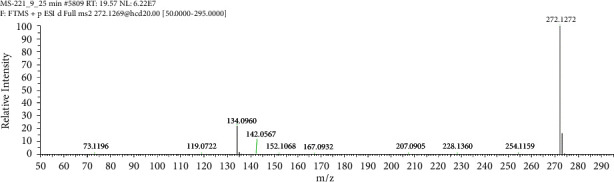
MS^2^ spectra of *m/z* 272.1272.

**Figure 9:**
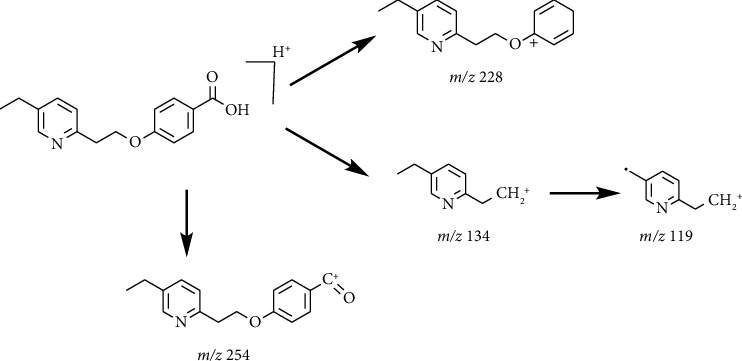
Fragmentation patterns of [M + H]^+^ for Impurity-2.

The HRMS spectrum of Impurity-2 showed a protonated molecular ion peak at *m/z* 272.1272 [M + H]^+^ corresponding to the molecular formula C_16_H_17_NO_3_ (exact mass: 271.1203).  Figure 8 shows the MS^2^ spectrum of *m/z* 272.1272. Characteristic product ions of Impurity-2 at *m/z* 134, 119, and 228 indicated that its structure is unchanged compared with pioglitazone except 5-methylene-2,4-thiazolodinedione unit. The characteristic product ions at *m/z* 254 and 228 were the loss of 18 Da and 44 Da from protonated molecular ion of Impurity-2, respectively, indicating that the structure of Impurity-2 contains carboxyl group (-COOH). The structure of Impurity-2 is shown in Table 2.  Figure 9 shows fragmentation patterns of [M + H]^+^ for Impurity-2.

Intermediate-1 may be oxidized into Impurity-2 under alkaline conditions. Thus, Impurity-2 is a process impurity.

**Figure 10:**
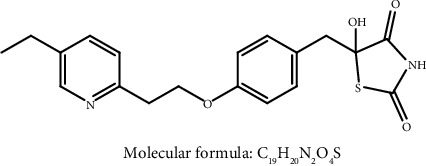
Chemical structures of impurity A listed in European Pharmacopeia 10.0.

**Figure 11:**
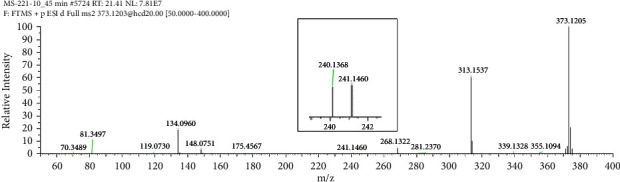
MS^2^ spectra of *m/z* 373.1205.

**Figure 12:**
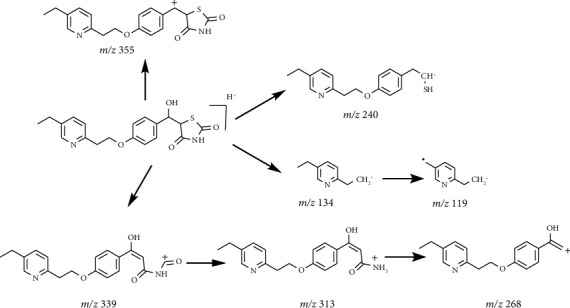
Fragmentation patterns of [M + H]^+^ for Impurity-3.

The HRMS analysis of Impurity-3 displayed a protonated molecular ion peak at *m/z* 373.1205 [M + H]^+^ which is compatible to the molecular formula C_19_H_20_N_2_O_4_S (exact mass: 372.1138) which has the same molecular formula of impurity A (Figure 10) listed in European Pharmacopeia 10.0. The retention time of Impurity-3 was earlier than impurity A in the chromatogram (Figure 2).  Figure 11 shows the MS^2^ spectrum of *m/z* 373.1205. The existence of characteristic product ions at *m/z* 134, 119, and 240 demonstrated that the structure of Impurity-3 is consistent with pioglitazone besides 5-methylene-2,4-thiazolodinedione unit. The characteristic product ion at *m/z* 355 was the loss of 18 Da from protonated molecular ion of Impurity-3, which showed the presence of a hydroxyl (-OH) group. Therefore, substituted position of the hydroxyl group was altered comparing with impurity A. The structure of Impurity-3 is shown in Table 2.  Figure 12 shows fragmentation patterns of [M + H]^+^ for Impurity-3.

**Figure 13:**
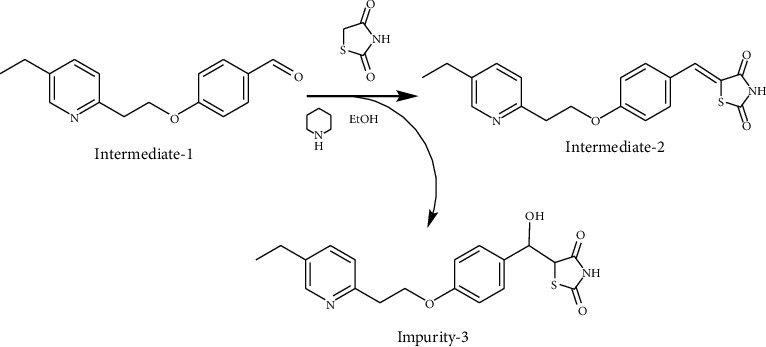
The formation mechanism for Impurity-3.

According to the mechanism of Knoevenagel condensation reaction, Impurity-3 was the by-product of the synthetic process of Intermediate-2 (Figure 13). Therefore, it belongs to process impurity.

**Figure 14:**
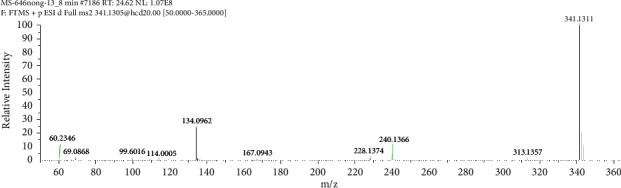
MS^2^ spectra of *m/z* 341.1311.

**Figure 15:**
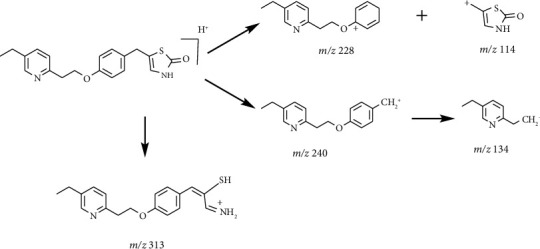
Fragmentation patterns of [M + H]^+^ for Impurity-4.

A protonated molecular ion peak at *m/z* 341.1311 [M + H]^+^ obtained in the HRMS spectrum of Impurity-4 was consistent with the molecular formula C_19_H_19_N_2_O_2_S (exact mass: 340.1240).  Figure 14 shows the MS^2^ spectrum of *m/z* 341.1311. The exact mass of Impurity-4 was 18 less than pioglitazone, which denoted the loss of H_2_O. Furthermore, the distinction between Impurity-4 and pioglitazone was the 2,4-thiazolodinedione unit owing to the presence of typical fragment ions at *m/z* 134 and 240. Therefore, a loss of H_2_O occurred on the 2,4-thiazolodinedione. The presence of a fragment ion at *m/z* 114 manifested that the thiazole ring became more stable corresponding to the increased stability by the formation of carbon-carbon double bond after the dehydration of the C-4 position carbonyl. The structure of Impurity-4 is shown in Table 2.  Figure 15 shows fragmentation patterns of [M + H]^+^ for Impurity-4.

**Figure 16:**
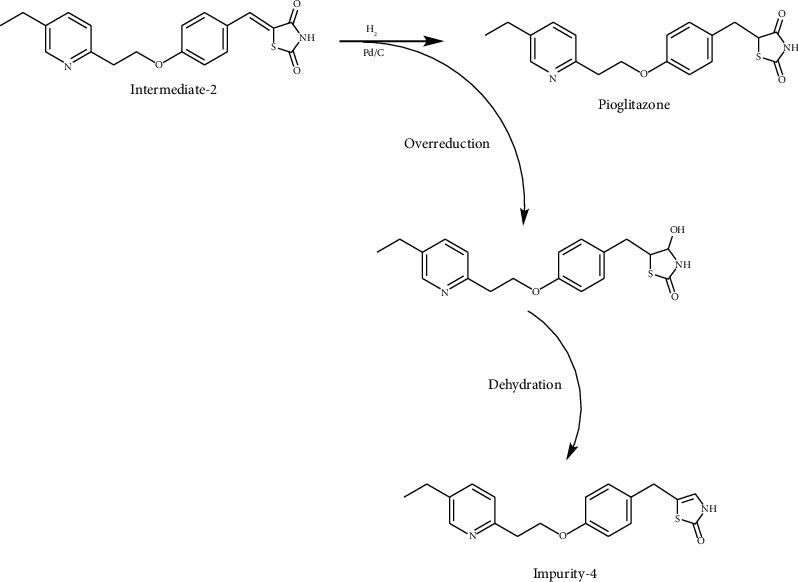
The formation mechanism for Impurity-4.

During the process of Intermediate-2 being reduced to pioglitazone, it may be over-reduced and then dehydrated to form Impurity-4 (Figure 16). Therefore, Impurity-4 is a process impurity.

**Figure 17:**
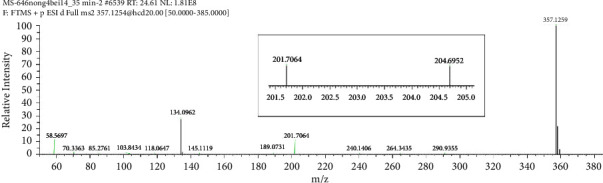
MS^2^ spectra of *m/z* 357.1259.

**Figure 18:**
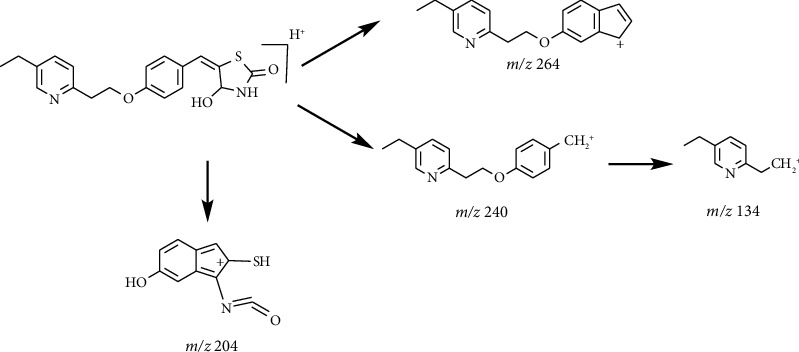
Fragmentation patterns of [M + H]^+^ for Impurity-5.

The HRMS data of Impurity-5 showed a protonated molecular ion peak at *m/z* 357.1259 [M+H]^+^ corresponding to the molecular formula C_19_H_20_N_2_O_3_S (exact mass: 356.1189), and this showed that Impurity-5 is the isomer of pioglitazone.  Figure 17 shows the MS^2^ spectrum of *m/z* 341.1311. The presence of fragment ions at *m/z* 134 and 240 indicated that its structure is unchanged compared to pioglitazone except 5-methylene-2,4-thiazolodinedione unit. The possible structure of Impurity-5 is deduced (Table 2) by referring the synthetic route of pioglitazone.  Figure 18 shows fragmentation patterns of [M+H]^+^ for Impurity-5.

**Figure 19:**
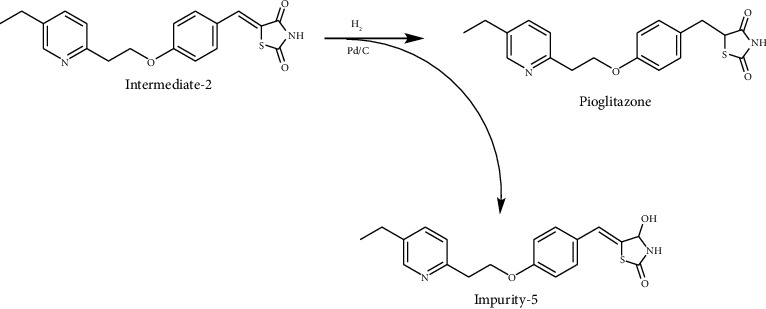
The formation mechanism for Impurity-5.

Impurity-5 is a process impurity. The 4-position carbonyl group of the 2,4-thiazolodinedione ring might be reduced in the process of Intermediate-2 reduction (Figure 19).

### 3.3. Toxicity Prediction of Target Impurities

As exhibited in Table 3, the genotoxicity of all target impurities belonged to Class 5 which was defined as “no structural alerts or alerting structure with sufficient data to demonstrate lack of mutagenicity or carcinogenicity” [14].

The value of TOX_Risk predicted by ADMET Predictor™ indicated the number of potential toxicity problems that a compound might have. The predicted compounds with the value below 3.3 are considered as safe. Table 3 shows that the values TOX_Risk of five target impurities are all less than 3.3. Overall, five target impurities were predicted as safe compounds.

## 4. Conclusions

The online desalting technique achieved by heart-cutting 2D-LC coupled with HRMS showed several advantages in structural identification of impurities. First, the aimed impurity can be transported into mass spectrometry without changing the mobile phase of the analytical method which may contain nonvolatile salt or high concentration salt. In addition, HRMS can offer information of accurate mass and secondary fragment ions which are helpful for structural elucidation. In this study, fragmentation patterns of pioglitazone and five unidentified impurities were investigated and applied to obtain structural information of these impurities. Two impurities, Impurity-1 and Impurity-4, were reported previously, whereas the remaining three were first reported in this article. The toxicity assessments of these five impurities were predicted, which indicated that they all have a good safety profile. This study may provide a reference for the quality control of pioglitazone hydrochloride preparations.

## Figures and Tables

**Table 1:** The gradient elution program of the second dimension.

Run time (min)	A% (%)	B% (%)	
0	95	5	Equilibrate column in second dimension
*t*	95	5	

*t* + 8	95	5	Online desalting step

*t* + 23	5	95	Transfer the desalted impurities into MS
*t* + 28	5	95	

*t* + 29	95	5	Re-equilibrate column

*t*: the time of target impurities is completely trapped into the two-dimensional separation system.

**Table 2:** Molecular formula, accurate mass, and elucidated structure of target impurities.

Impurity	Molecular formula	[M + H]^+^/(*m/z*)		Deviation (ppm)	Elucidated structure
		Theoretical	Experimental		
Impurity-1	C_16_H_19_NO_2_	258.1489	258.1480	3.5	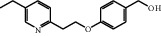
Impurity-2	C_16_H_17_NO_3_	272.1281	272.1272	3.3	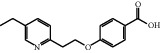
Impurity-3	C_19_H_20_N_2_O_4_S	373.1216	373.1205	2.9	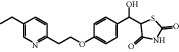
Impurity-4	C_19_H_20_N_2_O_2_S	341.1318	341.1311	2.1	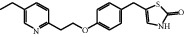
Impurity-5	C_19_H_20_N_2_O_3_S	357.1267	357.1259	2.2	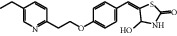

**Table 3:** The results of toxicity prediction of target impurities.

Impurity	Structure	TOX_Risk	TOX_Code	Derek prediction	Sarah prediction	ICH M7 class
Impurity-1	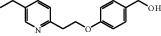	0		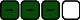	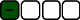	Class 5
Impurity-2	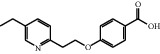	0		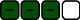	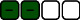	Class 5
Impurity-3	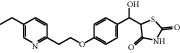	1	Xr	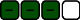	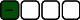	Class 5
Impurity-4	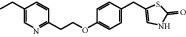	1	Xr	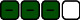	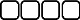	Class 5
Impurity-5	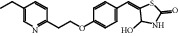	2	Xr, Hp	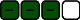	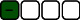	Class 5

ADMET Predictor™ provided predictions of TOX_Risk and TOX_Code; Xr: carcinogenicity in rat; Hp: hepatotoxicity.

## Data Availability

The datasets used to support the findings of this study are included within the article.
